# Phenotypic and molecular spectra of patients with switch/sucrose nonfermenting complex-related intellectual disability disorders in Korea

**DOI:** 10.1186/s12920-021-01104-9

**Published:** 2021-10-27

**Authors:** Yena Lee, Yunha Choi, Go Hun Seo, Gu-Hwan Kim, Changwon Keum, Yoo-Mi Kim, Hyo-Sang Do, Jeongmin Choi, In Hee Choi, Han-Wook Yoo, Beom Hee Lee

**Affiliations:** 1 Department of Pediatrics, Asan Medical Center Children’s Hospital, University of Ulsan College of Medicine, 88, Olympic-ro 43-gil, Songpa-gu, Seoul, 05505 Republic of Korea; 2 3Billion Inc., Seoul, Republic of Korea; 3 Medical Genetics Center, Asan Medical Center Children’s Hospital, University of Ulsan College of Medicine, Seoul, Republic of Korea; 4 Department of Pediatrics, Chungnam National University Sejong Hospital, College of Medicine, Chungnam National University, Sejong, Republic of Korea; 5 Genome Research Center for Birth Defects and Genetic Diseases, Asan Institute for Life Sciences, Asan Medical Center, Seoul, Republic of Korea; 6 Department of Genetic Counseling, University of Ulsan College of Medicine, Seoul, Republic of Korea

**Keywords:** Intellectual disability, Chromatin assembly and disassembly, Language development disorders, Corpus callosum, Whole-exome sequencing, Germline mutation, Phenotype

## Abstract

**Background:**

The switch/sucrose nonfermenting (SWI/SNF) complex is an adenosine triphosphate-dependent chromatin-remodeling complex associated with the regulation of DNA accessibility. Germline mutations in the components of the SWI/SNF complex are related to human developmental disorders, including the Coffin–Siris syndrome (CSS), Nicolaides–Baraitser syndrome (NCBRS), and nonsyndromic intellectual disability. These disorders are collectively referred to as SWI/SNF complex-related intellectual disability disorders (SSRIDDs).

**Methods:**

Whole-exome sequencing was performed in 564 Korean patients with neurodevelopmental disorders. Twelve patients with SSRIDDs (2.1%) were identified and their medical records were retrospectively analyzed.

**Results:**

*ARID1B*, found in eight patients, was the most frequently altered gene. Four patients harbored pathogenic variants in *SMARCA4*, *SMARCB1*, *ARID2*, and *SMARCA2.* Ten patients were diagnosed with CSS, and one patient without a typical phenotype was diagnosed with *ARID1B*-related nonsyndromic intellectual disability. Another patient harboring the *SMARCA2* pathogenic variant was diagnosed with NCBRS. All pathogenic variants in *ARID1B* were truncating, whereas variants in *SMARCA2*, *SMARCB1*, and *SMARCA4* were nontruncating (missense). Frequently observed phenotypes were thick eyebrows (10/12), hypertrichosis (8/12), coarse face (8/12), thick lips (8/12), and long eyelashes (8/12). Developmental delay was observed in all patients, and profound speech delay was also characteristic. Agenesis or hypoplasia of the corpus callosum was observed in half of the patients (6/12).

**Conclusions:**

SSRIDDs have a broad disease spectrum, including NCBRS, CSS, and *ARID1B*-related nonsyndromic intellectual disability. Thus, SSRIDDs should be considered as a small but important cause of human developmental disorders.

## Background

The switch/sucrose nonfermenting (SWI/SNF) complex, first purified from yeast, is an adenosine triphosphate (ATP)-dependent chromatin-remodeling complex that regulates DNA accessibility by mobilizing nucleosomes in an ATP-dependent manner [1]. The components of the SWI/SNF complex were first recognized as tumor-suppressor genes implicated in oncogenesis [2]. The association between this chromatin-remodeling complex and human developmental disorders was discovered and studied with remarkable progress using next-generation sequencing [3–5].

Coffin–Siris syndrome (CSS, MIM #135900) is characterized by intellectual disability (ID) and is often accompanied by a coarse face, hypertrichosis, sparse scalp hair, and hypoplasia/aplasia of the distal phalanx or nail of the fifth digit. After the discovery of *ARID1B*, several other genes (e.g., *ARID1A*, *SMARCA4*, *SMARCB1*, *SMARCE1*, *SOX11*, *ARID2*, and *DPF2*) were identified as the causative genes for CSS [6–11].

The Nicolaides–Baraitser syndrome (NCBRS, MIM #601358) overlaps with the CSS, with more severe ID associated with a dysmorphic coarse face, microcephaly, seizures, and prominence of the interphalangeal joints. This syndrome is caused by *SMARCA2*, which is also one component of the SWI/SNF complex [12].

As pathogenic variants in the SWI/SNF complex are continuously detected in more patients with ID, these conditions are considered as manifestations of one clinical continuum, with *ARID1B*-related ID and mild CSS at one end, more severe forms of CSS in the middle, and NCBRS at the other end of the spectrum [13]. Therefore, the concept of SWI/SNF complex-related intellectual disability disorders (SSRIDDs) was introduced to explain this clinical spectrum [13, 14].

This study analyzed 12 unrelated Korean patients with SSRIDDs confirmed via genetic testing while evaluating the cause of neurodevelopmental delay in these patients. Clinical information and the result of molecular analysis were analyzed to better characterize the phenotypic spectrum of SSRIDDs among Asian populations.

## Methods

### Subjects and clinical assessment

Whole-exome sequencing (WES) was used to evaluate 564 patients with neurodevelopmental disorders, such as developmental delay (DD), ID, epilepsy, neuromuscular disease, and central nervous system (CNS) anomalies, at the Medical Genetic Center of the Asan Medical Children’s Hospital, Seoul, Korea, from March 2018 to October 2020. If any candidate variants were found, parental genetic testing using Sanger sequencing was performed to verify the pathogenicity of the identified variants. Patients harboring pathogenic variants or microdeletions in the components of the SWI/SNF complex were analyzed in this study.

Clinical data were retrospectively collected to describe the detailed phenotypes of SSRIDDs. Standard deviation scores (SDSs) of the height and body weight were calculated based on the Korean National Growth Charts for children and adolescents [15]. Short stature was defined as the height SDS below − 2.0 SDS for age- and sex-matched normative data [15]. The degree of ID was assessed with an intelligence quotient (IQ) test in patients aged ≥ 5 years. IQ scores of 50–70 were considered to indicate mild ID, IQ scores of 35–50 were considered to indicate moderate ID, and IQ scores < 35 were considered to indicate severe ID. Developmental status indicated by the developmental quotient (DQ) was evaluated using the Korean infant and child development test (KICDT) [16], which was developed by the Development Education Enacting Subcommittee of the Korean Pediatrics Academy. KICDT was designed to assess development in five functional domains: gross motor, fine motor, social-personal, language, and cognitive-adaptive skills. DQ [DQ = (developmental age/chronological age) × 100] lower than 80 was regarded as abnormal development.

All subjects were born from nonconsanguineous Korean parents. Blood or buccal smear samples were obtained with the informed consent of the patients’ parents. This study was approved by the Institutional Review Board for Human Research of the Asan Medical Center (2021-0347).

### Molecular analysis

WES was performed using genomic DNA isolated from either whole blood or buccal epithelial cells. Exons of human genes (approximately 22,000) were captured using a SureSelect kit (version C2; Agilent Technologies, Inc., Santa Clara, CA, USA). The captured genomic regions were sequenced using a NovaSeq platform (Illumina, San Diego, CA, USA). Raw genome-sequencing data analyses involved alignment to the reference sequence [National Center for Biotechnology Information genome assembly GRCh37; accessed in February 2009]. Mean read depth was 100-fold, with 99.2% coverage higher than tenfold. Variant calling, annotation, and prioritization were performed as previously described [17].

Allele frequency of the general population was assessed using the Genome Aggregation Database (gnomAD; http://gnomad.broad institute.org/). The pathogenicity of the variants was evaluated following the guidelines of the American College of Medical Genetics and Genomics (ACMGG) [18]. In silico analysis was performed using prediction softwares, such as Polyphen-2 (http://genetics.bwh.harvard.edu/pph2/), MutationTaster (http://www.mutationtaster.org/), SIFT (https://sift.bii.a-star.edu.sg/), and PROVEAN (http://provean.jcvi.org/index.php).

Chromosomal microarray (CMA) was performed using the CytoScan 750 K assay platform (Thermo Fisher Scientific, Waltham, MA, USA). The genomic DNA (250 ng) extracted from the peripheral blood was digested using NspI and amplified using ligation-mediated polymerase chain reaction (PCR). The PCR product was purified, quantified, fragmented using DNase I, labeled with biotin, and hybridized overnight (16–18 h) in a CytoScan 750 K array. After hybridization, the sample was washed and stained with streptavidin using GeneChip Fulidics Station 450. Moreover, the array was scanned using GeneChip Scanner 3000 to generate a CEL file. The CEL file was analyzed using Chromosome Analysis Suite (Thermo Fisher Scientific) and converted to a CYCHP file to visualize the status of the genomic copy number and absence of heterozygosity.

## Results

Among the 564 patients with neurologic disorders, 12 patients had SSRIDDs (12/564 patients; 2.13%).

### Clinical features of patients with SSRIDDs

The clinical features of the 12 patients (7 females and 5 males) are described in Tables 1 and 2.

**Table 1:** Basic clinical information on the patients with SSRIDDs

Case ID	1	2	3	4	5	6	7	8	9	10	11	12
Diagnosis	CSS	CSS	A-ID	CSS	CSS	CSS	CSS	CSS	CSS	CSS	CSS	NCBRS
Sex	F	F	M	F	M	M	F	F	F	M	F	M
Age at initial visit	18 months	11 months	6 months	At birth	8 months	At birth	2 years	19 days	78 days	4 months	1 months	22 months
Reason of genetic testing	DD	DD	DD	DD	DD	DD, CNS anomaly	DD	DD	DD, epilepsy	DD	DD, short stature	DD, epilepsy
Age at diagnosis (year)	3	3	2	3	5	3	2	5	7	11 months	5	3
Current age (year)	3	6	3	4	6	3	3	6	8	1	15	3
GA at birth (weeks)	38	39	41	38	38	38	37	39	37	37	41	37
Birth weight (kg)	2.7	2.92	2.73	2.13	2.2	2.48	2.9	2.61	2.11	2.28	2.5	2.9
SGA	No	No	Yes	Yes	Yes	Yes	No	No	Yes	Yes	Yes	No
Perinatal event	OH	No	No	OH	TTN	No	No	No	Seizure	TTN	No	No
Current height (SDS)	− 1.33	0.37	− 1.13	− 2.8	− 3.02	− 3.51	0.21	− 2.72	− 2.17	− 3.57	− 1.97	− 0.01
Current weight (SDS)	− 0.16	− 0.27	− 0.62	− 2.49	− 2.19	− 2.96	− 0.45	− 2.32	− 3.1	− 4.06	− 1.59	0.24
Short stature	No	No	No	Yes	Yes	Yes	No	Yes	Yes	Yes	Yes (GH)	No

SSRIDDs, switch/sucrose nonfermenting complex-related intellectual disability disorders; CSS, Coffin–Siris syndrome; A-ID, *ARID1B*-related intellectual disability; NCBRS, Nicolaides–Baraitser syndrome; F, female; M, male; DD, developmental delay; CNS, central nervous system; GA, gestational age; SGA, small for gestational age; OH, oligohydramnios; TTN, transient tachypnea of the newborn; SDS, standard deviation score; GH, growth hormone

**Table 2:** Clinical features of patients with SSRIDDs

Case ID	1	2	3	4	5	6	7	8	9	10	11	12	Total
Diagnosis	CSS	CSS	A-ID	CSS	CSS	CSS	CSS	CSS	CSS	CSS	CSS	NCBRS	
Sex	F	F	M	F	M	M	F	F	F	M	F	M	
*Dysmorphic features*													
Microcephaly	No	No	No	No	Yes	No	No	No	Yes	Yes	Yes	No	4/12
Coarse face	Yes	Yes	No	Yes	No	Yes	Yes	Yes	Yes	No	No	Yes	8/12
Spares hair	No	No	No	No	No	Yes	Yes	No	No	Yes	Yes	No	4/12
Hypertrichosis	Yes	Yes	No	Yes	Yes	No	Yes	Yes	Yes	No	No	Yes	8/12
Narrow forehead	No	Yes	No	No	No	No	No	No	No	Yes	No	No	2/12
Thick eyebrow	Yes	No	Yes	Yes	No	Yes	Yes	Yes	Yes	Yes	Yes	Yes	10/12
Long eyelashes	Yes	No	Yes	Yes	No	Yes	Yes	No	Yes	Yes	No	Yes	8/12
Eyes	Prominent	–	–	Long PF	–	Prominent	–	Puffy eyes	EF, Down slanting PF	Short PF	EF, HT	HT	
Flat & broad nasal bridge	No	No	No	Yes	Yes	No	Yes	No	Yes	Yes	Yes	No	6/12
Low-set ears	Yes	No	No	Yes	No	No	Yes	No	Yes	Yes	Yes	No	6/12
Philtrum	–	–	–	Short	–	–	Short	–	Short	–	Long	–	
Large mouth	No	No	No	Yes	No	No	No	No	Yes	No	Yes	No	3/12
Thick lips	Yes	No	No	Yes	No	Yes	Yes	Yes	Yes	No	Yes	Yes	8/12
Micrognathia	Yes	No	No	Yes	No	Yes	Yes	No	Yes	Yes	Yes	No	7/12
Hypoplastic terminal phalanx of the 5th finger	No	No	No	No	No	Yes	Yes	No	Yes	No	No	No	3/12
Hypoplastic nail	No	No	No	Yes	No	Yes	Yes	No	No	Yes	No	Yes	5/12
Clinodactyly	No	No	No	Yes	Yes	No	No	No	No	No	No	No	2/12
*Congenital anomalies*													
CHD	PFO	PFO	Normal	ASD	Normal	Normal	Normal	Normal	Normal	Normal	VSD	Normal	4/12
GI system	–	CP	–	FD	FD	FD	–	CP	IH	IH, FD	IH, FD	–	
Cryptorchidism	–	–	No	–	No	No	–	–	–	Yes	–	Yes	2/5
Laryngomalacia	No	Yes	No	Yes	No	No	No	No	Yes	Yes	No	No	4/12
Frequent infections	No	No	Yes	Yes	Yes	Yes	Yes	Yes	Yes	No	No	No	7/12
Agenesis/hypoplasia of CC	No	No	ND	Yes	No	Yes	Yes	Yes	Yes	Yes	ND	No	6/12
CNS anomaly	Small pons, ARC	Normal	ND	Hypoplasia of OB	Normal	Mega cisterna magna	No	No	No	No	ND	Normal	
Hearing loss	No	No	No	No	Yes	No	No	No	No	Yes	No	No	2/12

SSRIDDs, switch/sucrose nonfermenting complex-related intellectual disability disorders; CSS, Coffin–Siris syndrome; A-ID, *ARID1B*-related intellectual disability; NCBRS, Nicolaides–Baraitser syndrome; F, female; M, male; PF, palpebral fissure; EF, epicanthal folds; HT, hypertelorism; CHD, congenital heart defect; PFO, patent foramen ovale; ASD, atrial septal defect; VSD, ventricular septal defect; GI, gastrointestinal; CP, constipation; FD, feeding difficulty; IH, inguinal hernia; CC, corpus callosum; ND, no data; CNS, central nervous system; ARC, arachnoid cyst; OB, olfactory bulb

Ten patients were clinically diagnosed with CSS, and one patient with subtle dysmorphic features and mild ID was diagnosed with *ARID1B*-related nonsyndromic ID. Moreover, one patient harboring a *SMARCA2* mutation was diagnosed with NCBRS.

The mean age at diagnosis was 39.4 ± 18.9 months. Genetic testing was performed for all patients to evaluate the cause of DD (12/12 patients, 100%), which was combined with epilepsy (2/12 patients, 16.7%), short stature (1/12 patients, 8.3%), or a CNS anomaly (1/12 patients, 8.3%).

Six patients (6/12 patients, 50%) were born small for their gestational age. In addition, five patients had an abnormal perinatal history, including oligohydramnios (2/12 patients, 16.7%), transient tachypnea of the newborn (2/12 patients, 16.7%), and neonatal seizures (1/12 patients, 8.3%). The mean height at the latest evaluation (age, 5.1 ± 3.5 years) was − 1.80 ± 1.36 SDS, and the mean body weight was − 1.66 ± 1.33 SDS. Seven patients (7/12 patients, 58.3%) were observed to have short stature.

Frequently observed dysmorphic features were thick eyebrows (10/12 patients, 83.3%), hypertrichosis (8/12 patients, 66.7%), coarse face (8/12 patients, 66.7%), thick lips (8/12 patients, 66.7%), and long eyelashes (8/12 patients, 66.7%). A broad nasal bridge and low-set ears were found in six patients (6/12 patients, 50%). Hypoplastic nail and terminal phalanx of the fifth finger, which are characteristic features of CSS, were found in five (5/12 patients, 41.7%) and three patients (3/12 patients, 25%), respectively. A congenital heart defect was identified in four patients (4/12 patients, 33.3%). Several patients had gastrointestinal problems, including feeding difficulties during infancy (5/12 patients, 41.7%), inguinal hernia (3/12 patients, 25%), and constipation (2/12 patients, 16.7%). Frequent upper and lower respiratory tract infections were noted in seven patients (7/12 patients, 58.3%). Two of the five male patients had cryptorchidism (2/5 patients, 40%). Agenesis or hypoplasia of the corpus callosum was observed in half of the patients (6/12 patients, 50%).

DD/ID was a cardinal feature (Table 3). Hypotonia during infancy associated with gross motor delay was noted in all patients (12/12 patients, 100%). The mean age at walking without assistance was 20.4 ± 3.7 months. All patients had a delay in language development, including four patients with no meaningful speech at all (4/12 patients, 33.3%). The degree of ID was assessed in patients aged > 5 years. Two patients had mild ID (2/12 patients, 16.7%), whereas three had moderate ID (3/12 patients, 25%). Seizure and hyperactivity were documented in five (5/12 patients, 41.7%) and four patients (4/12 patients, 33.3%), respectively.

**Table 3:** Degree of DD/ID in patients with SSRIDDs

Case ID	1	2	3	4	5	6	7	8	9	10	11	12	Total
Diagnosis	CSS	CSS	A-ID	CSS	CSS	CSS	CSS	CSS	CSS	CSS	CSS	NCBRS	
*Developmental quotient (DQ)*^a^													
Age at test (years)	1.9	4.4	2.4	2.7	0.75	2.4	3.2	4.6	5.3	ND	ND	3	
Cognitive-adaptive	56.5	–	82.8	68.8	66.7	72.4	57.9	43.6	26.6			33.3	8/9
Language	34.8	20.8	75.9	53.1	55.6	41.4	42.1	38.2	–			16.7	9/9
Social-personal	47.8	34.0	75.9	62.5	55.6	51.7	47.4	40	–			16.7	9/9
Fine motor	69.6	58.5	75.9	75	44.4	69.0	57.9	40	–			41.7	9/9
Gross motor	65.2	37.7	69.0	68.8	44.4	58.6	52.6	38.2	42.2			66.7	10/10
*Follow-up evaluation*													
Age (years)	3	6	3	4	6	3	3	6	8	1	15	3	
Degree of ID	–	Mild	–	–	Moderate	–	–	Moderate	Moderate	–	Mild (IQ 69)	–	
Age of walking alone (months)	18	20	19	23	24	24	24	20	ND	ND	ND	12	
Language delay	No speech	No speech	Yes	Yes	Yes	No speech	Yes	Yes	Yes	Yes	Yes	No speech	12/12
Hyperactivity	No	Yes	No	No	Yes	Yes	No	Yes	No	No	No	No	4/12
Autistic features	No	Yes	No	No	No	No	No	No	No	No	No	No	1/12
Seizure	No	No	No	No	Yes	No	No	Yes	Yes	Yes	No	Yes	5/12

DD, developmental delay; ID, intellectual disability; SSRIDDs, switch/sucrose nonfermenting complex-related intellectual disability disorders; CSS, Coffin–Siris syndrome; A-ID, *ARID1B*-related intellectual disability, NCBRS, Nicolaides–Baraitser syndrome; ND, no data

^a^DQ was measured using the Korean infant and child development test (KICDT), and a score lower than 80 was regarded to indicate developmental delay

### Molecular analysis of patients with SSRIDDs

WES identified 10 pathogenic variants in 10 patients, which neither parent carried. All of these ten patients had a confirmed de novo mutation origin. No pathogenic variants were observed using WES in the remaining two patients (subjects 5 and 11), whereas further analysis using CMA revealed microdeletions at regions encompassing the genes of the SWI/SNF complex (Table 4).

**Table 4:** Genotypes of patients with SSRIDDs (*ARID1B*: NM_020732.3, *SMARCA4*: NM_001128845.1, *SMARCB1*: NM_001007468.2, *SMARCA2*: NM_003070.5)

ID	Gene	Diagnosis	Nucleotide change	Amino acid change	Exon	Inheritance	Known mutation	Interpretation
1	*ARID1B*	CSS	c.1311C > G	p.Tyr437*	1	De novo	Novel	Pathogenic
2	*ARID1B*	CSS	c.1612C > T	p.Gln538*	2	De novo	Known	Pathogenic
3	*ARID1B*	A-ID	c.2362C > T	p.Gln788*	7	De novo	Known	Pathogenic
4	*ARID1B*	CSS	c.2692C > T	p.Arg898*	9	De novo	Known [8]	Pathogenic
5	*ARID1B*	CSS	arr 6q25.3 (157,482,390_157,561,632) × 1, 34 kb deletion		Deletion from exon 10 to 18^a^	ND^b^	Novel	Pathogenic
6	*ARID1B*	CSS	c.3345 + 1G > A	–	Intron 12	De novo	Novel	Pathogenic
7	*ARID1B*	CSS	c.4849C > T	p.Gln1617*	18	De novo	Novel	Pathogenic
8	*ARID1B*	CSS	c.5725del	p.Gln1909Lysfs*65	20	De novo	Novel	Pathogenic
9	*SMARCA4*	CSS	c.3128G > T	p.Arg1043Leu	22	De novo	Novel	Likely pathogenic
10	*SMARCB1*	CSS	c.1087A > G	p.Lys363Glu	8	De novo	Known	Pathogenic
11	*ARID2*	CSS	arr 12q12-13.11 (43,005,992_46,669,000) × 1, 3.7 Mb deletion		Haploinsufficiency	De novo	Known [19]	Pathogenic
12	*SMARCA2*	NCBRS	c.3479C > G	p.Ala1160Gly	25	De novo	Novel	Pathogenic

SSRIDDs, switch/sucrose nonfermenting complex-related intellectual disability disorders; CSS, Coffin–Siris syndrome; A-ID, *ARID1B*-related intellectual disability, NCBRS, Nicolaides–Baraitser syndrome

^a^Multiplex ligation-dependent probe amplification confirmed a microdeletion from exons 10 to 18 of *ARID1B*

^b^ND, No data. Parental genetic testing was not performed

Ten patients harbored missense, nonsense, or frameshift mutations in the SWI/SNF complex. *ARID1B* was the most common causative gene (8/12 patients, 66.7%). Four pathogenic variants in *ARID1B* (p.Tyr437*, c.3345 + 1G > A, p.Gln1617*, and p.Gln1909Lysfs*65) were novel, whereas the other three variants in *ARID1B* (p.Gln538*, p.Gln788*, and p.Arg898*) had been previously reported (https://www.ncbi.nlm.nih.gov/clinvar/variation/374179/, https://www.ncbi.nlm.nih.gov/clinvar/variation/450773/, and [8]). Pathogenic variants in *ARID1B* were either nonsense, frameshift, or splicing-site mutations. All pathogenic variants in *ARID1B* were distributed throughout the entire exon, and no mutational hotspots were noted. All variants in *ARID1B* were interpreted as pathogenic according to the ACMGG guidelines [18]. In subject 5, CMA revealed a 34-kb deletion at 6q25.3 (chr6: 157,482,390–157,561,632 [hg19]). Further evaluation using multiplex ligation-dependent probe amplification confirmed a microdeletion from exons 10 to 18 of *ARID1B*.

The remaining three patients harbored mutations in the other components of the SWI/SNF complex (i.e., *SMARCA4*, *SMARCB1*, and *SMARCA2*)*.*

A novel variant in *SMARCA4* (p.Arg1043Leu) was identified in subject 9, which was absent from the general population database (gnomAD). This variant was predicted to be “disease causing” in MutationTaster, “damaging” in SIFT, and “deleterious” in PROVEAN. A missense change at this amino acid residue, SMARCA4 p.Arg1043Trp, was previously reported as a likely pathogenic variant in ClinVar. Therefore, *SMARCA4* c.3128G > T (p.Arg1043Leu) was interpreted as a likely pathogenic variant based on the evidence of PS2, PM2, PM5, and PP3.

SMARCB1 p.Lys363Glu observed in subject 10 was previously reported (https://www.ncbi.nlm.nih.gov/clinvar/variation/212263/). Consequently, it was considered as a pathogenic variant following the addition of PS2 after confirming the de novo mutation origin (PS2, PM2, PP2, PP3, and PP5).

SMARCA2 p.Ala1160Gly observed in subject 12, who was diagnosed with NCBRS, was located in a mutational hotspot (C-terminal helicase domain) and absent from the general population database. In silico analysis predicted this variant to be “probably damaging” in PolyPhen-2, “disease causing” in MutationTaster, and “damaging” in SIFT. Thus, SMARCA2 p.Ala1160Gly was classified as a pathogenic variant (PS2, PM1, PM2, PP2, and PP3).

In Subject 11, CMA revealed a de novo 3.7-Mb deletion at the chromosomal region 12q12-13.11, which caused the entire *ARID2* gene to be deleted (chr12: 43,005,992–46,669,000 [hg19]), causing *ARID2* haploinsufficiency [19].

## Discussion

This study provided clinical and molecular information on 12 Korean patients with SSRIDDs. These 12 patients were recruited from the neurodevelopmental disorder cohort who underwent WES or CMA for elucidating the genetic cause of their condition. *ARID1B*, identified in eight patients, was the most frequently altered gene in this study. The remaining four patients harbored pathogenic variants or microdeletions in *SMARCA4*, *SMARCB1*, *SMARCA2*, and *ARID2*. The clinical diagnoses were CSS for 10 patients, *ARID1B*-related nonsyndromic ID for one patient, and NCBRS for one patient.

Among the patients in the neurodevelopmental disorder cohort, 2.13% had SSRIDDs (12/564, 2.13%). Unexplained ID due to SWI/SNF complex mutations was estimated to be up to 3%, and the data (2.13%) of this study supported this idea [20]. Hoyer et al. [3] reported that *ARID1B* mutations were identified in 0.9% of unexplained ID cases.

A definite genotype–phenotype correlation could not be established owing to the small number of patients. However, several phenotypic differences were found among various genotypes.

*ARID1B* mutations are considered to be the leading cause of CSS (68–83%) [7, 8, 21]. In this study, the pathogenic variants in *ARID1B* were identified in 66.7% of patients (8/12 patients). Clinical phenotypes associated with *ARID1B* alterations have been reported to be highly variable and not severe compared to phenotypes of other genotypes [22]. As the use of broad genetic tests such as WES is becoming widespread, individuals who may not fit the diagnosis of classic CSS but rather present with more inconclusive phenotypes are now being discovered. These patients with *ARID1B*-associated ID are expanding the phenotypic spectrum of the *ARID1B*-related disorder. The major differences between *ARID1B*-ID and *ARID1B*-CSS are the presence of typical dysmorphic features, including thick eyebrows, long eyelashes, hypoplastic/absent nail or distal phalanx of the fifth finger, and hypertrichosis [23].

For example, subject 3 was incidentally found to have a pathogenic variant in *ARID1B* during the evaluation of his mild DD. At the first examination, no dysmorphic features were noted in subject 3. However, the patient was reevaluated after identifying a pathogenic variant in *ARID1B*, and thick eyebrows and long eyelashes were noted. However, his phenotype was not sufficient to make a clinical diagnosis of CSS.

The patients with *ARID1B*-associated CSS in this study were likely to have a coarse face, hypertrichosis, thick eyebrows, large mouths, thick lips, long eyelashes, and micrognathia. Nail hypoplasia and/or a short distal phalanx of the fifth finger, which are known as cardinal CSS features, were identified in three patients (subjects 4, 6, and 7).

Previous studies [7, 8, 21] reported that a hypoplastic nail or a short distal phalanx of the fifth finger are present in 50%–68% of patients. According to a web-based survey (www.arid1bgene.com), which is an open collection of clinical information on patients with *ARID1B* mutations, the incidences of a hypoplastic fifth fingernail and short distal phalanx of the fifth finger were estimated to be 24.6% (42/171 patients) and 22.0% (37/168 patients), respectively. Previously reported high incidences (50–68%) of these abnormalities may reflect an ascertainment bias because *ARID1B* mutations were preferentially sought after among those with clinically diagnosed CSS [7, 8, 21]. In the present study, three (subjects 4, 6, and 7) out of seven patients with *ARID1B*-associated CSS (3/7 patients, 42.9%) exhibited nail and/or distal phalanx abnormalities, which corroborated the previously reported data (48%) [22].

The position of the pathogenic variants in *ARID1B* may not influence the severity of the clinical phenotypes. Santen et al. [24] found no relationship between the variant position on cDNA and clinical severity. For example, patients who had pathogenic variants in exon 20, at the 3′ terminal region of the gene, had severe ID [24]. Among the present cases, subject 8, who had a variant in exon 20, had short stature, moderate ID, and classical features of CSS.

Almost all patients with genetic alterations in *SMARCA4* were reported to have hirsutism, thick eyebrows, long eyelashes, and a less coarse face [25]. Subject 9, with a pathogenic variant in *SMARCA4*, also exhibited these typical features.

The pathogenic variants in *SMARCB1* lead to a severe form of CSS with various CNS anomalies and severe growth retardation [7, 8]. Subject 10 harbored a *SMARCB1* variant in exon 8, which is a highly-conserved region and well-established causative domain for CSS [7, 8]. Considered small for gestational age at birth, the patient underwent gastrostomy due to severe feeding difficulties. Severe growth retardation and microcephaly were also observed. Brain magnetic resonance imaging at 6 months revealed partial agenesis of the corpus callosum.

Subject 11 had mild ID with a profound short stature. As previously described [19], the patient exhibited both RASopathy-related features (e.g., profound short stature, epicanthal folds, down slanting palpebral fissures, and webbed neck) and CSS-like phenotypes (e.g., thick eyebrows, thick upper lips, and a large mouth). CMA revealed a 3.7-Mb deletion at chromosome 12q12-13.11 causing complete deletion of *ARID2.* As one of the components in the SWI/SNF complex, *ARID2* haploinsufficiency has been shown to be associated with CSS-like phenotypes [10]. A previous study demonstrated increased extracellular signal-regulated kinase (ERK) activation in ARID2 haploinsufficiency, suggesting an association between the SWI/SNF complex and RAS–MAPK pathway [19].

Subject 12, with the *SMARCA2* variant, displayed typical features of NCBRS (e.g., coarse face with hypertrichosis, thick eyebrows, thick lips, long eyelashes, nail hypoplasia, and microcephaly), but did not have prominent interphalangeal joints. Cognitive dysfunction was more severe in this patient than in those with other types of SSRIDDs. Differential diagnosis is sometimes confusing because CSS and NCBRS are overlapping syndromes that share similar phenotypes. Moreover, the clinical diagnosis may change according to the results of molecular analysis [8, 13]. Molecular confirmation is thus required to make an accurate diagnosis between these two overlapping syndromes.

Similar to previous studies [13, 21], variants in *ARID1B* in this study were truncating (nonsense or splicing-site mutations), whereas those in *SMARCA4*, *SMARCB1*, and *SMARCA2* were nontruncating (missense mutation). The *ARID1B* haploinsufficiency is a pathogenic mechanism that leads to CSS or *ARID1B*-related ID. Subject 5 with an exon 10–18 deletion in *ARID1B* also showed a CSS phenotype. *AIRD2* haploinsufficiency seems to have caused a CSS-like phenotype as well as ID in subject 12. All variants in *SMARCA4*, *SMARCB1*, and *SMARCA2* were missense mutations, implying that they may exert a gain-of-function or dominant-negative mechanism of pathogenicity [13, 21].

The SWI/SNF complex components were initially recognized as tumor-suppressor genes associated with oncogenesis. Inactivating mutations in several SWI/SNF components have recently been identified in a wide variety of tumors, including rhabdoid and lung cancer tumors [26]. Furthermore, truncating and missense germline mutations in *SMARCB1* and truncating germline mutations in *SMARCA4* have been shown to lead to a cancer predisposition syndrome [27, 28]. Several cases with tumor formation were found among patients with SSRIDDs. Papillary thyroid cancer was reported in a patient with an interstitial 6q25 deletion, including *ARID1B* [29]. Moreover, a patient carrying an *ARID1A* pathogenic variant with hepatoblastoma was described previously in the literature [6]. van der Sluijs et al. [23] reported a boy with an *ARID1B* variant diagnosed with a Sertoli–Leydig cell tumor and a temporal glioneuronal tumor at 3 and 12 years, respectively. Longer observational periods are needed to conclude whether there is an association between SSRIDDs and cancer predisposition.

The limitation of this study should be noted. As a retrospective study, some clinical information was not available for some patients. The phenotypes among the patients were variable because of their varying ages. Thus, a longer observational period and larger patient population are needed to determine the complete clinical features and disease courses of these patients.

## Conclusions

SSRIDDs can be found in a small but considerable proportion of the neurodevelopmental disorder patient cohort. Some common clinical features (e.g., hypertrichosis, coarse face, thick eyebrows, long eyelashes, and thick lips) and agenesis or hypoplasia of the corpus callosum can be clues suggesting SSRIDDs. Moreover, SSRIDD seems to be a disorder spectrum with *ARID1B*-related ID on one end, classic CSS in the middle, and NCBRS on the other end [22]. The phenotypic spectrum of SSRIDDs will be more clearly documented as more individuals with SSRIDDs are identified with large-scale genomic analysis of unselected patient cohorts and followed up for a longer term.

## Data Availability

All data supporting the presented results are included in this published article. The raw data of whole-exome sequencing of the patient in this study are not publicly available to protect participant confidentiality, but they are available from the corresponding author on reasonable request. Please contact Professor BH Lee at the Department of Medical Genetics in the Asan Medical Center Children’s hospital for any requests to access the data. Reference sequences for *ARID1B* (NC_000006.12), *SMARCA4* (NC_000019.10), *SMARCB1* (NC_000022.11), *SMARCA2* (NC_000009.12), and *ARID2* (NC_000012.12) are available in the GenBank repository. The links to the GenBank repositories are as follows; *ARID1B* (https://www.ncbi.nlm.nih.gov/nuccore/NC_000006.12?from=156776026&to=157210779&report=genbank), *SMARCA4* (https://www.ncbi.nlm.nih.gov/nuccore/NC_000019.10?from=10960999&to=11062277&report=genbank), *SMARCB1* (https://www.ncbi.nlm.nih.gov/nuccore/NC_000022.11?from=23786966&to=23838009&report=genbank), *SMARCA2* (https://www.ncbi.nlm.nih.gov/nuccore/NC_000009.12?from=2015347&to=2193624&report=genbank), and *ARID2* (https://www.ncbi.nlm.nih.gov/nuccore/NC_000012.12?from=45729706&to=45908037&report=genbank). Databases used in this study were Human Gene Mutation Database (HGMD, http://www.hgmd.cf.ac.uk), ClinVar database (https://www.ncbi.nlm.nih.gov/clinvar), gnomAD Browser (https://gnomad.broadinstitute.org/), SIFT (http://provean.jcvi.org/index.php), PROVEAN (http://provean.jcvi.org/index.php), PolyPhen-2 (http://genetics.bwh.harvard.edu/pph2/), and MutationTaster (http://www.mutationtaster.org/).

## Abbreviations

ACMGG: American College of Medical Genetics and Genomics
ATP: Adenosine triphosphate
CMA: Chromosomal microarray
CNS: Central nervous system
CSS: Coffin–Siris syndrome
DD: Developmental delay
ID: Intellectual disability
NCBRS: Nicolaides–Baraitser syndrome
PCR: Polymerase chain reaction
SDS: Standard deviation scores
SSRIDD: Switch/sucrose nonfermenting complex-related intellectual disability disorder
SWI/SNF: Switch/sucrose nonfermenting
WES: Whole-exome sequencing
